# Measurement of the efficacy of 2% lipid in reversing bupivacaine- induced asystole in isolated rat hearts

**DOI:** 10.1186/1471-2253-14-60

**Published:** 2014-07-30

**Authors:** Hongfei Chen, Yun Xia, Binbin Zhu, Xiawei Hu, Shihao Xu, Limei Chen, Thomas J Papadimos, Wantie Wang, Quanguang Wang, Xuzhong Xu

**Affiliations:** 1 Department of Anesthesiology, The First Affiliated Hospital of Wenzhou Medical University, 2 Fuxue Road, 325000 Zhejiang, China; 2 Department of Pathophysiology, Wenzhou Medical University, Wenzhou, Zhejiang, China; 3 Department of Anesthesiology, The Ohio State University Medical Center, Ohio, USA

**Keywords:** Anaesthetics local-bupivacaine, Complications-cardiac arrest, Lipid emulsion

## Abstract

**Background:**

The reversal efficacy of 2% lipid emulsion in cardiac asystole induced by different concentrations of bupivacaine is poorly defined and needs to be determined.

**Methods:**

Forty-two male Sprague–Dawley rats were randomly divided into seven groups: B40, B60, B80, B100, B120, B140 and B160, n = 6. The Langendorff isolated heart perfusion model was used, which consisted of a balanced perfusion with Krebs-Henseleit solution for 25 minutes and a continuous infusion of 100 μmol/L bupivacaine until asystole had been induced for 3 minutes. The hearts in the seven groups were perfused with Krebs-Henseleit solution containing a 2% lipid emulsion, and 40, 60, 80, 100, 120, 140 or 160 μmol/L bupivacaine, respectively. Cardiac recovery was defined as a spontaneous and regular rhythm with a rate-pressure product > 10% of the baseline value for more than 1 minute. Our primary outcome was the rate-pressure product 25 minutes after cardiac recovery. Other cardiac function parameters were also recorded.

**Results:**

All groups demonstrated cardiac recovery. During the recovery phase, heart rate, rate-pressure product, the maximum left ventricular pressure rise and decline in heart rate in the B120-B160 groups was significantly lower than those in the B40-B80 groups (*P* < 0.05). The concentration of bupivacaine and the reversal effects of a 2% lipid emulsion showed a typical transoid S-shaped curve, R^2^ = 0.9983, IC_50_ value was 102.5 μmol/L (95% CI: 92.44 - 113.6).

**Conclusions:**

There is a concentration-response relationship between the concentrations of bupivacaine and the reversal effects of 2% lipid emulsion.

## Background

Rapid infusion of a lipid emulsion to treat cardiotoxicity induced by bupivacaine, as originally proposed by Weinberg et al. [1] is now well established [2-8]. Although the mechanism of lipid emulsion has not yet been fully elucidated, it is generally accepted that a “lipid sink” resulting from the lipid emulsion causes bupivacaine to be removed from the serum [1,4,9].

Recently, the use of a rapid infusion of lipid emulsion for the management of severe local anaesthestic toxcity has been incorporated into the 2010 safety guidelines of the Association of Anaesthetists of Great Britain and Ireland (AAGBI) [10]. These guidelines indicate that the maximum dose of a 20% lipid emulsion is 12 ml/kg, with a theoretical plasma concentration of about 2.9%. Meanwhile, the American Society of Regional Anesthesia and Pain Medicine (ASRA) guidelines recommend a maximum dose of 10 ml/kg, with a theoretical plasma concentration of about 2.5% [11,12]. Subsequent to these guidelines Chen et al. [13] demonstrated that a 2% lipid emulsion was an effective resuscitative concentration when added to an isolated rat heart perfusate consisting of 40 μmol/L bupivacaine. However, the reversal efficacy of 2% lipid emulsions for higher concentrations of bupivacaine in the circulation has not been studied.

Here we report the measurement of the reversal efficacy of a 2% lipid emulsion in cardiac asystole induced by varied concentrations of bupivacaine, and demonstrate a concentration-response relationship between these varied concentrations and the reversal efficacy of the 2% lipid emulsion. Our primary outcome was the calculation of the rate-pressure product (RPP) after resuscitation of bupivacaine-induced asystole in isolated rat hearts using a model that varied the concentrations of bupivacaine. We also examined the secondary outcomes of heart rate (HR), left ventricular developed pressure (LVdevP = left ventricular systolic pressure - left ventricular end-diastolic pressure), and the maximum rates of left ventricular pressure change (±dP/dt_max_) that resulted from the resuscitation.

## Methods

The study was conducted with the approval of Wenzhou Medical College’s Animal Care and Use Committee (Zhejiang, China). The entire study was performed at the Wenzhou Medical College.

### Experimental animals

Forty two male Sprague–Dawley rats, weighing 280 ~ 330 g were provided by the animal center of Wenzhou Medical College (Zhejiang, China).

### Preparation of isolated heart perfusion model

The rats were anesthetized with a 350 mg/kg chloral hydrate intraperitoneal injection, followed by anticoagulation with an injection of 1000 U/Kg heparin via the inferior vena cava. After rapid thoracotomy coring, a Langendorff device (ML870B2, AD Instruments, Australia) was applied to establish retrograde irrigation under constant temperature (37°C) and pressure (120 mmHg). The rat hearts were then perfused with Krebs-Henseleit (K-H) solution (NaCl 118 mmol/L, KCl 4.7 mmol/L, MgSO_4_ 1.2 mmol/L, KH_2_PO_4_ 1.2 mmol/L, NaHCO_3_ 25.0 mmol/L, CaCl_2_ 2.5 mmol/L, glucose 10 mmol/L, pH 7.40 ± 0.05). The perfusate was exposed to 95% O_2_ and 5% CO_2_ for 30 min. Spontaneously beating hearts were warmed using an insulation cover. The left ventricular pressure was continuously monitored by a latex balloon placed in the left ventricle. Saline was intermittently injected into the balloon to maintain the left ventricular end-diastolic pressure (LVEDP) at 4–10 mmHg.

Data were collected by a PowerLab biological signal processing and analysis system (ML870, Australia Ed Instruments) and Chart 5.5.6 biological signal recording software. Parameters of cardiac function were recorded by placement of a copper wire electrode into the right atrial and apical epicardium, respectively, and an additional reference electrode was placed on the aorta. The isolated hearts were then perfused with K-H solution for 25 minutes, and when cardiac functioned stabilized further experimental interventions proceeded.

### Experimental grouping and treatments

Forty-two male Sprague–Dawley rats were randomly divided into seven groups each perfused with a different bupivacaine concentration (in μmol/L): B40, B60, B80, B100, B120, B140 B160, with n = 6 in each group. The Langendorff isolated heart perfusion model was used. This consisted of a balanced perfusion with K-H solution for 25 min (baseline time, or time zero, was designated as Tb), and a continuous infusion of 100 μmol/L bupivacaine (Bupivacaine hydrochloride powder, batch number 100959032, Sigmar, USA) until asystole had been induced for 3 minutes [14,15]. The hearts in the seven groups were then perfused with a K-H solution containing a 2% lipid emulsion (20% Intralipid, Huarui Pharmaceutical Co., Ltd., Suzhou, China), and 40, 60, 80, 100, 120, 140, or 160 μmol/L bupivacaine, respectively.

When an isolated rat heart recovered its ability to beat, the perfusate was continued for an additional 25 minutes (to the final time designated as Te). In our study cardiac recovery was defined as the presence of a spontaneous heartbeat accompanied by a regular heart rhythm with a RPP > 10% of the baseline value for more than 1 minute.

### Outcomes measured

We compared the time from initiation of the bupivacaine infusion to asystole (designated Ts) and the time from the end of the 100 μmol/L bupivacaine infusion to cardiac recovery (designated Tr) in all groups. The following cardiac function parameters were recorded or calculated: heart rate (HR), left ventricular developed pressure (LVdevP = left ventricular systolic pressure - left ventricular end-diastolic pressure), rate-pressure product (RPP = HR * LVdevP), and the maximum rates of left ventricular pressure rise and fall (±dP/dt_max_). The ratio of the highest RPP (RPPh) during the recovery to baseline RPP (RPPr), and the ratio of RPP at Te to baseline RPP (RPPe) were also documented.

### Time of measurements

In all groups, cardiac function parameters were recorded at baseline (Tb), and at 1 (T1), 5 (T5), 10 (T10), 15 (T15), 20 (T20) and 25 (Te) minutes after cardiac recovery.

### Concentration-response curve fitting

The logarithm values of bupivacaine concentration were placed along the abscissa and the corresponding RPPr were placed on the vertical axis to provide a fit for the concentration-response curve. Non-linear regression was used to analyze and verify whether the concentration-effect relationship existed.

### Statistical analysis

SPSS was the statistical software used (version 14.0, Chicago, IL). Measurement data were tested for normality. Continuous variables were presented as means ± SD. Weight, Ts, Tr, RPPr, RPPe and other non-continuous data were analyzed by one-way ANOVA, and we applied the LSD test for pairwise comparison when significance was achieved. Continuous cardiac function parameters among groups were compared by repeated-measures of analysis of variance, and the Bonferroni correction was used for further multiple comparisons. Statistical significance was considered as *P* < 0.05. The relationship between bupivacaine concentration and the reversal efficacy of the 2% lipid emulsion was fitted in non-linear fashion using GraphPad Prism 5.0 software (GraphPad software, San Diego, CA).

## Results

A total of forty-two rats were included in the statistical analysis, with n = 6 in each group.

### Baseline values

There were no differences in baseline weight and cardiac function parameters among the seven groups in the study.

### Time to asystole (Ts) and time to recovery (Tr)

All hearts developed asystole after a 100 μmol/L bupivacaine infusion. Ts did not vary among groups (Table 1). All isolated hearts in the seven groups exhibited cardiac recovery. Tr in the B40-B80 groups was shorter than those in the B120-B160 groups (*P* < 0.05, Table 1).

**Table 1:** The results of Ts, Tr, RPPr and RPPe for all groups

**Group**	**Ts (second)**	**Tr (second)**	**RPPr (100%)**	**RPPe (100%)**
B40	39 ± 7	34 ± 14	55 ± 10	37 ± 10
B60	40 ± 10	44 ± 12^*†^	50 ± 14^*^	29 ± 9^*^
B80	40 ± 8	62 ± 11^*†^	43 ± 7^*^	18 + 3^*†^
B100	39 ± 9	74 ± 10^*†^	33 ± 8^*†‡^	15 ± 4^*†^
B120	38 ± 11	80 ± 88^*†‡^	22 ± 5^*†‡^	8 ± 3^*†‡^
B140	42 ± 7	81 ± 6^*†‡^	16 ± 3^*†‡^	5 ± 1^*†‡^
B160	38 ± 4	84 ± 10^*†‡^	13 ± 3^*†‡^	4 ± 2^*†‡^

All values are given as mean ± SD, n = 6 for each group. Ts = the time from initiation of bupivacaine infusion to asystole, Tr = the time since the end of bupivacaine infusion to cardiac recovery, RPPr = the ratio of the maximum rate-pressure product during recovery to baseline value, RPPe = the ratio of the rate-pressure product at the end of infusion time to baseline value.

^*^P < 0.05, compared with B40 group.

^†^P < 0.05, compared with B60 group.

^‡^P < 0.05, compared with B80 group.

### Cardiac function parameters

The isolated hearts in all bupivacaine groups demonstrated cardiac recovery after being infused with a 2% lipid emulsion (although the extent of recovery varied among groups).

**Figure 1:**
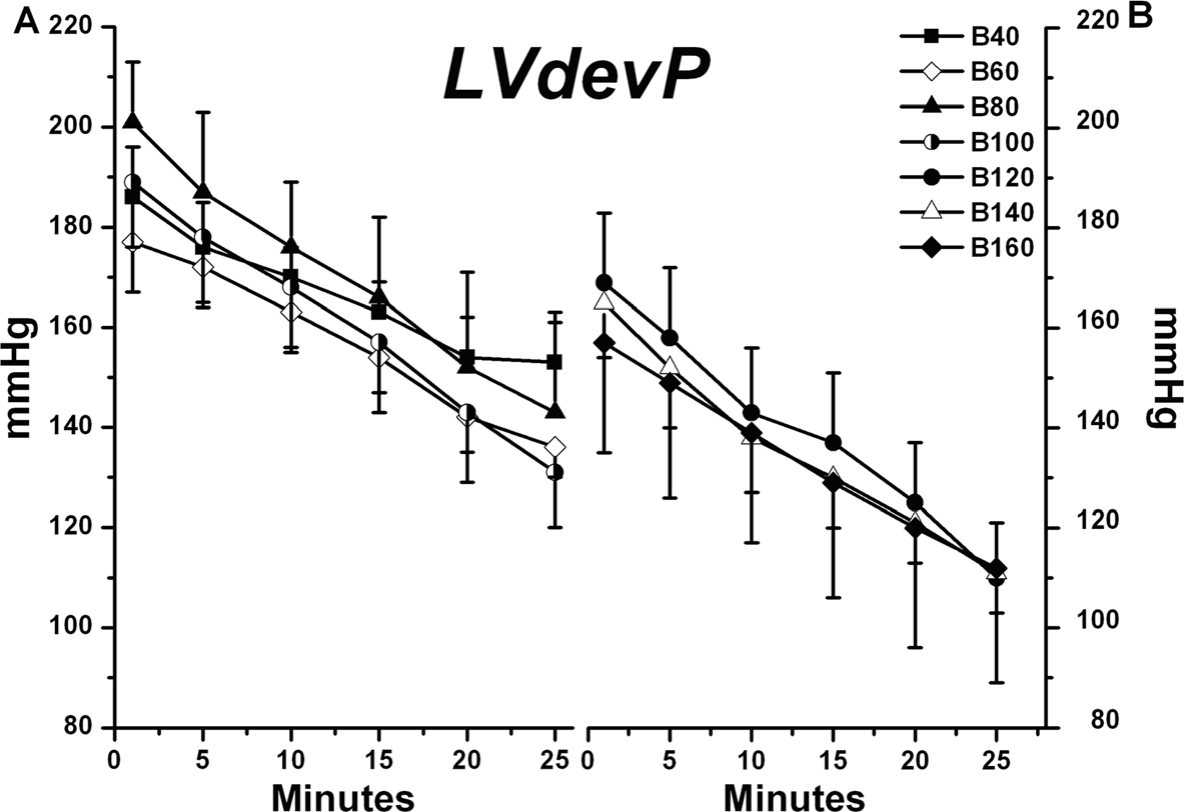
**Left ventricular developed pressure (LVdevP) in B40 – B100 groups (A) and B120 – B160 groups (B) are shown during recovery from bupivacaine-induced asystole (mean ± SD; n = 6 for all values).** LVdevp → contractility of isolated rat hearts. *P* values are as follows: B120 *vs.* B40, *P* = 0.026; B140 *vs.* B40, *P* = 0.005; B160 *vs.* B40, *P* = 0.002; B120 *vs.* B80, *P* = 0.005; B140 *vs.* B80, *P* = 0.001; B160 *vs.* B80, *P* < 0.001; B140 *vs.* B100, *P* = 0.049; B160 *vs.* B100, *P* = 0.023.

During the recovery phase, LVdevP in the B40 group was greater than that in the B120-B160 groups (*P* < 0.05), (Figure 1).

**Figure 2:**
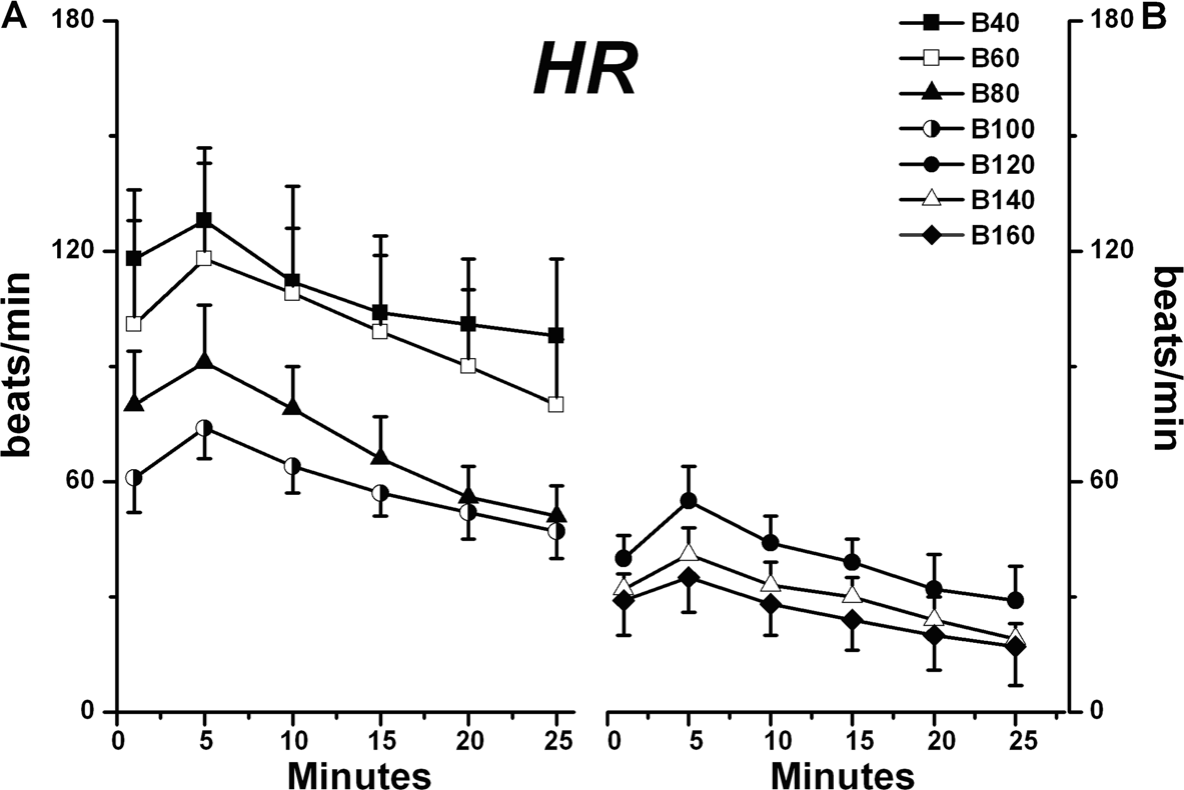
**Heart rate (HR) in B40 – B100 groups (A) and B120 – B160 groups (B) are shown during recovery from bupivacaine-induced asystole (mean ± SD; n = 6 for all values).** HR → conduction of isolated rat hearts. *P* values are as follows: B80, B100, B120, B140, B160 *vs.* B40, *P* < 0.001; B80 *vs.* B60, *P* = 0.006; B100, B120, B140, B160 *vs.* B60, *P* < 0.001; B120 *vs.* B80, *P* = 0.003; B140, B160 *vs.* B80, *P* < 0.001; B140 *vs.* B100, *P* = 0.005; B160 *vs.* B100, *P* = 0.001.

In regard to HR, the B40-B60 groups achieved a higher HR than the B80-B160 groups (*P* < 0.01). The B80 group exceeded B120-B160 groups (*P* < 0.01), and the B100 group exceeded the B140-B160 groups (*P* < 0.01, Figure 2). Furthermore, the maximum HR in all groups was achieved within 5 minutes after cardiac recovery, which were 128 ± 19, 118 ± 25, 91 ± 15, 74 ± 9, 55 ± 9 41 ± 7 and 35 ± 9 beats/min, respectively. The B40 group had the greatest recovery rate among all groups (45%).

**Figure 3:**
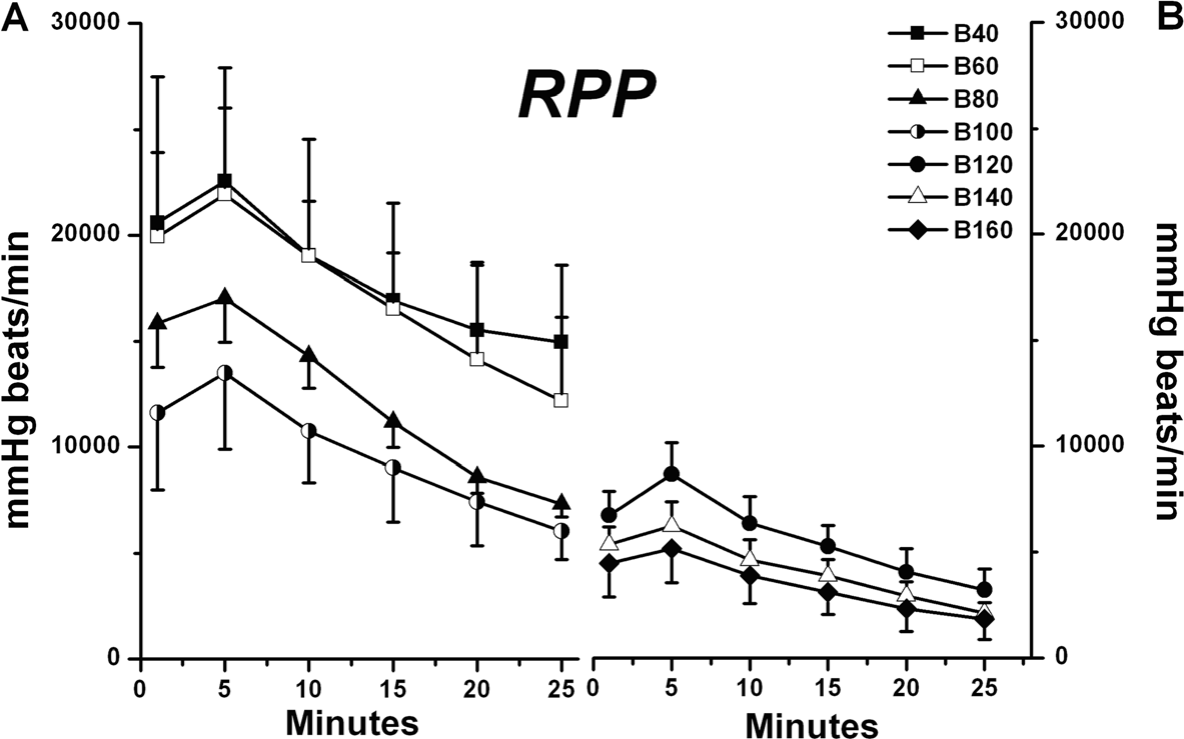
**Rate-pressure product (RPP) in B40 – B100 groups (A) and B120 – B160 groups (B) are shown during recovery from bupivacaine-induced asystole (mean ± SD; n = 6 for all values).** RPP → ventricular systolic function of isolated rat hearts. *P* values are as follows: B80 *vs.* B40, *P* = 0.008; B100, B120, B140, B160 *vs.* B40, *P* = 0.000; B100, B120, B140, B160 *vs.* B60, *P* = 0.000; B120 *vs.* B80, *P* = 0.002; B140, B160 *vs.* B80, *P* < 0.001; B140 *vs.* B100, *P* = 0.014; B160 *vs.* B100, *P* = 0.004.

In regard to RPP, the B40-B60 groups exceeded the B80-B160 groups (*P* < 0.05), and the B80-B100 groups exceeded the B140-B160 groups (*P* < 0.05, Figure 3). The maximum value of RPP in all groups was achieved within 5 minutes of cardiac recovery, these were 22551 ± 3460, 21228 ± 5546, 17025 ± 2067, 13496 ± 3611, 8690 ± 1473, 6220 ± 1139, 5236 ± 1544 mmHg•beats.min^−1^, respectively.

The -dP/dt_max_ in the B60 group was greater than that in the B160 group (*P* < 0.05), while -dP/dt_max_ in the B80 group was greater than in the B120-B160 groups (*P* < 0.05); +dP/dt_max_ in the B40-B100 groups were greater than in the B120-B160 groups (*P* < 0.05).

RPPr in all groups recovered within 5 minutes after cardiac recovery, which were 55, 50, 43, 33, 22, 16 and 13%, respectively (Table 1). RPPr in the B40-B60 groups were greater than those in the B100-B160 groups (*P* < 0.05), while RPPr in the B80-B100 groups were greater than in the B140-B160 groups (*P* < 0.05). Otherwise, the RPPe in the seven groups were 37, 29, 18, 14, 8, 5 and 4%, respectively, and the RPPe in the B40-B100 groups were greater than in the B120-B160 groups (*P* < 0.05, Table 1).

### Concentration - response relationship

**Figure 4:**
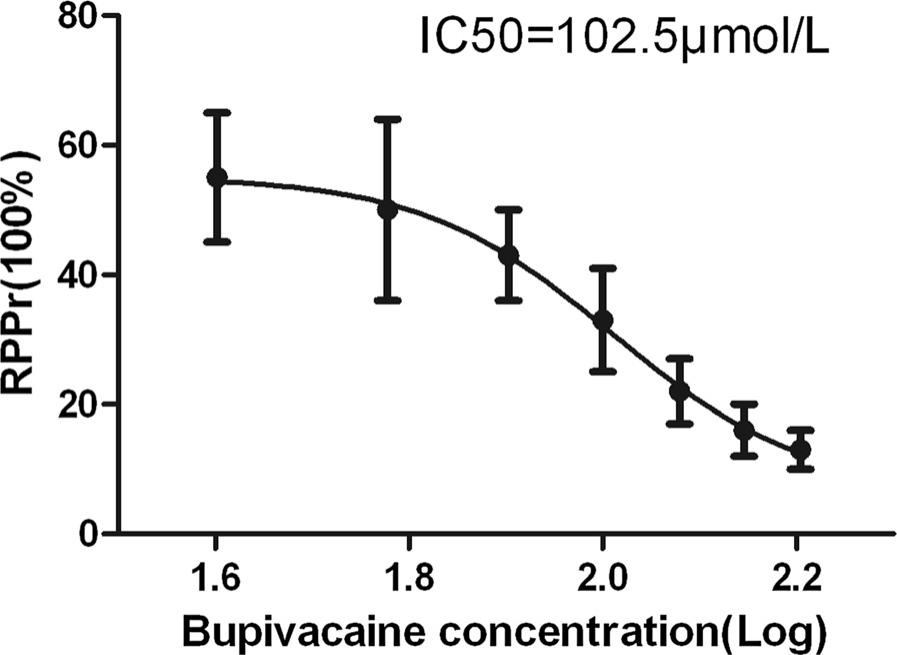
**The concentration - response curve of the increasing concentrations of bupivacaine and the effects of 2% lipid emulsion, in reverse of bupivacaine-induced asystole in isolated rat hearts.** RPPr = the ratio of the maximum rate-pressure product during recovery to baseline value.

The different concentrations of bupivacaine studied and their corresponding effect (RPPr) on the isolated rats hearts after cardiac recovery were fitted in a non-linear fashion using the equation, *y* = 0.065 + 0.487/[1 + 10^− 4.4 * (2 − *x*)^], which demonstrated a transoid S-shaped concentration response curve (Figure 4), with R2 = 0.9983. The curve demonstrated that the reversal efficacy of the 2% lipid emulsion decreased with an increasing concentration of bupivacaine. The pharmacological parameters obtained from the non-linear regression analysis were a minimum value of 6.5% and a maximum value of 55.2%, IC50 value = 102.5 μmol/L (95% CI 92.44 - 113.6), LogIC50 value = 2.0, HillSlope value = −4.4.

## Discussion

In our isolated rat heart model of bupivacaine-induced asystole, a 2% lipid emulsion reversed cardiac toxicity induced by 40–160 μmol/L of bupivacaine. With an increasing concentration of bupivacaine, the major cardiac function parameters including HR, RPP, RPPr, LVdevP and ± dP/dt_max_ declined in all groups. The concentration-response relationship showed a reverse S-shaped curve, with a 102.5 μmol/L IC_50_ value of bupivacaine. Cardiac recovery and function were much lower and the recovery times were much longer when the bupivacaine concentrations were above 100 μmol/L.

In our study, we used a 100 μmol/L bupivacaine infusion to induce asystole, and then added increasing concentrations of bupivacaine into the reperfusate to simulate different levels of bupivacaine-related cardiac toxicity. Liu et al. [14] reported a portion of isolated hearts in the control group that did not receive therapy with a lipid emulsion could indeed recover when the background concentration of bupivacaine was < 30 μmol/L. Previous to this, Chen et al. [13] reported that none of the isolated hearts in the control group could be resuscitated when the background concentration of bupivacaine was ≥ 40 μmol/L. Therefore, we studied the graduated increase of bupivacaine concentrations beyond 40 μmol/L to a maximum of 160 μmol/L in order to better assess its effects. Left ventricular systolic pressure was an important method to assess the cardiac contractility, and was highly related to HR. We used RPP as a parameter of cardiac recovery because it is a good indicator of the interaction between contractility and HR; we found that RPPr is the most useful indicator of cardiac recovery.

Our work differs from previous studies in that we have focused on the reversal efficacy of a 2% fat emulsion in attenuating the cardiac toxicity induced by different concentrations of bupivacaine. We found that the reversal efficacy of a 2% fat emulsion on the isolated heart model was significantly reduced when the concentration of bupivacaine in the reperfusion solution increased, especially when it exceeded 100 μmol/L. The concentration-response relationship in this study demonstrated a typical S-shaped curve when a bupivacaine concentration of 102.5 μmol/L IC_50_ was reached. Expectations that lower and/or higher lipid concentrations may shift our bupivacaine-response curve leftward or rightward are reasonable. However, such confirmation will require further study.

It is of interest that Ruan et al. [15] found that a 2% fat emulsion could reduce the concentration of bupivacaine by 20-40%. Therefore, our findings may not be explained by the mechanism of “Lipid sink” alone [1,4,9]; Other mechanisms may also involved in the recovery process [16-18].

Chen et al. [13] also found that lipid administration in bupivacaine-induced asystole displayed a time-response relationship in the RPP of isolated rat hearts. We confirmed their finding that RPP decreased over the time. In our study, RPP gradually decreased after achieving its maximum at 5 minutes. RPP in groups with bupivacaine concentrations > 100 μmol/L were all less than 10% of the baseline value at the end of perfusion (25 min), which suggested that these hearts had an ineffective cardiac recovery.

The clinically recommended maximum dose of bupivaicane is 175 mg [19]. If 175 mg of bupivacaine quickly enters into the circulation of a 70 kg patient (with blood volume about 7% of body weight), the theoretical value of the blood concentration of bupivacaine will be 35.7 μg/mL (104.2 μmol/L), a concentration that approaches 102.5 μmol/L (as described above). However, if the assumption is made that the bupivacaine entering the circulation will combine with plasma proteins [20], this may make the plasma concentration of free bupivacaine much lower. We surmise that under such conditions, to achieve the lipid concentration of 2% in the circulation, a treatment dose of 7 mL/kg may be sufficient.

Our study has several limitations. Our bupivacaine-induced asystole model of isolated rat hearts used only a single factor with which to influence the model. Whereas in real clinical scenarios, cardiac arrest is often accompanied by other factors such as hypoxia, acidosis, pulmonary edema and other perturbations [21-23], thus making the pathology more complicated. Furthermore, the applicability of our findings to other species (rabbits and swine for example, let alone humans) may not be valid and requires further experimental confirmation.

## Conclusion

Our study demonstrates that a 2% lipid emulsion can reverse 40–160 μmol/L concentrations of bupivacaine-induced asystole in isolated rat hearts. We have also demonstrated that there is a concentration-response relationship between the concentrations of bupivacaine and the reversal efficacy of a 2% lipid emulsion. A bupivacaine concentration of 102.5 μmol/L is the point at which the reversal efficacy of a 2% lipid emulsion will decline sharply. We encourage our colleagues to pursue further studies to better optimize the model we have proposed and any of its underlying assumptions.

## Abbreviations

IC_50_: Inhibitory concentration 50; Ts: Time to asystole; Tr: Time to recovery; HR: Heart rate; LVdevP: Left ventricular developed pressure; RPP: Rate-pressure product; ±dP/dt_max_: The maximum left ventricular pressure rise and fall rate.

## Competing interests

The authors declare that they have no competing interests.

## Authors’ contributions

All those listed as authors contributed to the preparation of the manuscript. HC: Study design, animal experiment, data collection, data analysis and writing up of the first draft of the paper. YX: Study design, data analysis and editing the paper. BZ, XH, SX: Study design, animal experiment and data collection. QW, LC: Study design and data analysis. WW: Study design. TP:Editing the paper. XX: Study design, data analysis and writing up of the first draft of the paper. All authors have read and approved the final version.

## Pre-publication history

The pre-publication history for this paper can be accessed here:

http://www.biomedcentral.com/1471-2253/14/60/prepub

## References

[B1] Weinberg GL VadeBoncouer T Ramaraju GA Garcia-Amaro MF Cwik MJ Pretreatment or resuscitation with a lipid infusion shifts the dose–response to bupivacaine-induced asystole in rats Anesthesiology 1998 88 1071 1075 9579517 10.1097/00000542-199804000-00028

[B2] Harvey M Cave G Chanwai G Nicholson T Successful resuscitation from bupivacaine-induced cardiovascular collapse with intravenous lipid emulsion following femoral nerve block in an emergency department Emerg Med Australas 2011 23 209 214 21489169 10.1111/j.1742-6723.2011.01401.x

[B3] Cordell CL Schubkegel T Light TR Ahmad F Lipid infusion rescue for bupivacaine- induced cardiac arrest after axillary block J Hand Surg [Am] 2010 35 144 146 10.1016/j.jhsa.2009.10.018 20117318

[B4] Rosenblatt MA Abel M Fischer GW Itzkovich CJ Eisenkraft JB Successful use of a 20% lipid emulsion to resuscitate a patient after a presumed bupivacaine-related cardiac arrest Anesthesiology 2006 105 217 218 16810015 10.1097/00000542-200607000-00033

[B5] Weinberg G Ripper R Feinstein DL Hoffman W Lipid Emulsion Infusion Rescues Dogs From Bupivacaine-Induced Cardiac Toxicity Reg Anesth Pain Med 2003 28 198 202 12772136 10.1053/rapm.2003.50041

[B6] Marwick PC Levin AI Coetzee AR Recurrence of cardiotoxicity after lipid rescue from bupivacaine-induced cardiac arrest Anesth Analg 2009 108 1344 1346 19299810 10.1213/ane.0b013e3181979e17

[B7] Admani B Essajee F Successful resuscitation of a three month old child with intralipid infusion, presumed to have bupivacaine induced seizures and cardiovascular complications: case report East Afr Med J 2010 87 354 356 23451560

[B8] Li Z Xia Y Dong X Chen H Xia F Wang X Dong H Jin Z Ding X Papadimos TJ Xu X Lipid Resuscitation of Bupivacaine Toxicity: Long-chain Triglyceride Emulsion Provides Benefits over Long- and Medium-chain Triglyceride Emulsion Anesthesiology 2011 115 1219 1228 22037638 10.1097/ALN.0b013e318238be73

[B9] Weinberg GL Ripper R Murphy P Edelman LB Hoffman W Strichartz G Feinstein DL Lipid infusion accelerates removal of bupivacaine and recovery from bupivacaine toxicity in the isolated rat heart Reg Anesth Pain Med 2006 31 296 303 16857549 10.1016/j.rapm.2005.02.011

[B10] AAGBI Safety Guideline Management of Severe Local Anesthetic Toxicity 2010 Available at: http://www.aagbi.org/publications/guidelines/docs/la_toxicity_2010.pdf. (Accessed December 2010)

[B11] Weinberg GL Treatment of local anesthetic systemic toxicity (LAST) Reg Anesth Pain Med 2010 35 189 193 10.1097/AAP.0b013e3181d246c3 20216036

[B12] Neal JM Bernards CM Butterworth JF 4th Di Gregorio G Drasner K Hejtmanek MR Mulroy MF Rosenquist RW Weinberg GL ASRA practice advisory on local anesthetic systemic toxicity Reg Anesth Pain Med 2010 35 152 161 20216033 10.1097/AAP.0b013e3181d22fcd

[B13] Chen Y Xia Y Liu L Shi T Shi K Wang Q Chen L Papadimos TJ Xu X Lipid emulsion reverses bupivacaine-induced asystole in isolated rat hearts: concentration-response and time-response relationships Anesthesiology 2010 113 1320 1325 21068661 10.1097/ALN.0b013e3181fc63ed

[B14] Liu L Xia Y Chen Y Wang Q Shi T Wang F Small RH Xu X The Comparative Effects of Lipid, Epinephrine, and Their Combination in the Reversal of Bupivacaine-Induced Asystole in the Isolated Rat Heart Anesth Analg 2012 114 886 893 21519055 10.1213/ANE.0b013e3182166a0a

[B15] Ruan W French D Wong A Drasner K Wu AH A mixed (long- and medium-chain) triglyceride lipid emulsion extracts local anesthetic from human serum in vitro more effectively than a long-chain emulsion Anesthesiology 2012 116 334 339 22273855 10.1097/ALN.0b013e318242a5f1

[B16] Weinberg GL Palmer JW VadeBoncouer TR Zuechner MB Edelman G Hoppel CL Bupivacaine inhibits acylcarnitine exchange in cardiac mitochondria Anesthesiology 2000 92 523 528 10691241 10.1097/00000542-200002000-00036

[B17] Partownavid P Umar S Li J Rahman S Eghbali M Fatty-acid oxidation and calcium homeostasis are involved in the rescue of bupivacaine-induced cardiotoxicity by lipid emulsion in rats Crit Care Med 2012 40 2431 2437 22647409 10.1097/CCM.0b013e3182544f48 PMC3401289

[B18] Mottram AR Valdivia CR Makielski JC Fatty acids antagonize bupivacaine-induced I(Na) blockade Clin Toxicol (Phila) 2011 49 729 733 21970771 10.3109/15563650.2011.613399

[B19] Rosenberg PH Veering BT Urmey WF Maximum recommended doses of local anesthetics: A multifactorial concept Reg Anesth Pain Med 2004 29 564 575 15635516 10.1016/j.rapm.2004.08.003

[B20] Tsen LC Tarshis J Denson DD Osathanondh R Datta S Bader AM Measurements of maternal protein binding of bupivacaine throughout pregnancy Anesth Analg 1999 89 965 968 10512273 10.1097/00000539-199910000-00027

[B21] Weinberg GL Di Gregorio G Ripper R Kelly K Massad M Edelman L Schwartz D Shah N Zheng S Feinstein DL Resuscitation with lipid versus epinephrine in a rat model of bupivacaine overdose Anesthesiology 2008 108 907 913 18431127 10.1097/ALN.0b013e31816d91d2

[B22] Hiller DB Gregorio GD Ripper R Kelly K Massad M Edelman L Edelman G Feinstein DL Weinberg GL Epinephrine impairs lipid resuscitation from bupivacaine overdose: a threshold effect Anesthesiology 2009 111 498 505 19704251 10.1097/ALN.0b013e3181afde0a

[B23] Di Gregorio G Schwartz D Ripper R Kelly K Feinstein DL Minshall RD Massad M Ori C Weinberg GL Lipid emulsion is superior to vasopressin in a rodent model of resuscitation from toxin-induced cardiac arrest Crit Care Med 2009 37 993 999 19237909 10.1097/CCM.0b013e3181961a12

